# Scope of an Integrative Neurophysiotherapy Approach in Achieving Gross Motor Milestones in a Child with Meningitis: A Case Report

**DOI:** 10.7759/cureus.49540

**Published:** 2023-11-28

**Authors:** Anam F Pathan, Nikita H Seth, Nishigandha P Deodhe

**Affiliations:** 1 Department of Neurophysiotherapy, Ravi Nair Physiotherapy College, Datta Meghe Institute of Higher Education and Research, Wardha, IND

**Keywords:** physiotherapy rehabilitation, subdural hematoma, hydrocephalus, seizures, meningitis

## Abstract

Meningitis caused by bacteria, which is an inflammation of the meninges affecting the pia, arachnoid, and subarachnoid space, is still one of the leading causes of death and morbidity in infants and young children. *Neisseria meningitidis*, group B streptococcus (GBS), *Haemophilus influenzae* type B (Hib), *Listeria monocytogenes*, and *Streptococcus pneumoniae* have been found to be the most frequent causative agents. Infants and children can have modest, fluctuating, non-specific, or even absent clinical signs of bacterial meningitis. They may include bulging fontanelles, vomiting, diarrhea, respiratory distress, hypothermia, lethargy, irritability, poor feeding, and fever in babies. In this case report, an 18-month-old child presented to a local hospital with complaints of multiple episodes of high-grade fever. After 10 days, his symptoms worsened and he experienced two episodes of seizures at one-day intervals at night. He was taken to Acharya Vinoba Bhave Rural Hospital for further management. Blood investigations revealed seropositive results for dengue virus infection. On MRI and CT scan, it was diagnosed as an old case of subdural hematoma in the right frontotemporal region of the brain. The patient was on intravenous ceftriaxone and phenytoin. Gross motor developmental milestones in children with meningitis can be improved with early integrative neurophysiotherapy and a goal-oriented therapeutic regimen that includes mobility exercises, proprioceptive neuromuscular facilitation techniques, positioning, oromotor retraining, neurodevelopmental techniques, and balance and coordination retraining. A complex case presents with bacterial meningitis, hydrocephalus, and seizure disorder. The bacterial infection inflames the protective membranes of the brain, causing hydrocephalus. Increased cerebrospinal fluid puts pressure on the brain, leading to seizures. Managing these interconnected conditions requires a multidisciplinary approach making it unique, involving infectious disease, neurology, and neurosurgery expertise.

## Introduction

Bacterial meningitis is an extremely serious condition caused by a bacterial infection that inflames the protective membranes surrounding the brain and spinal cord. It is a major cause of mortality and morbidity in newborns and children and requires immediate medical attention [1]. Depending on the age range, different microorganisms frequently cause bacterial meningitis [2]. The most common causative organisms have been identified as *Neisseria meningitidis*, group B streptococcus (GBS), *Haemophilus influenzae* type B (Hib), *Listeria monocytogenes*, and *Streptococcus pneumoniae*. Over 80% of pediatric cases of acute bacterial meningitis were brought on by these microorganisms [3]. Infants and children can have modest, fluctuating, non-specific, or even absent clinical signs of bacterial meningitis. They may include bulging fontanelles, vomiting, diarrhea, respiratory distress, hypothermia, lethargy, irritability, poor feeding, and fever in babies [4]. The course of the clinical manifestations of acute bacterial meningitis varies depending on the patient's age, but they typically take 24 to 48 hours to appear [5]. While meningeal signs and symptoms may not be obvious, seizures are the initial sign of meningitis in 16.7% of children and one-third of these individuals [6]. Short-term problems include seizures, focal neurological impairments, and subdural effusions, as well as long-term complications include hearing loss, cognitive impairment, hydrocephalus, learning disability, and epilepsy [7]. Around 7% of children who develop bacterial meningitis develop hydrocephalus [8]. A rise in intracranial pressure leading to an aberrant dilatation of the ventricles is a common feature of the pathologic condition known as hydrocephalus, which disrupts the dynamics of normal cerebrospinal fluid flow [9]. Communicating hydrocephalus, which occurs in up to 52% of cases, is the second most common type after obstructive hydrocephalus [10]. It may be necessary to install a temporary or permanent ventricular shunt depending on the severity of the hydrocephalus and the resultant neurologic impairment [11]. Achieving a better clinical outcome requires prompt surgery, accurate diagnosis, and appropriate antibiotic treatment [12].

## Case presentation

Patient information

An 18-month-old child presented to a local hospital with multiple high-grade fevers that were unresponsive to antibiotics and antipyretics. After 10 days, his symptoms worsened, and he experienced two episodes of seizures at one-day intervals at night. He was taken to Acharya Vinoba Bhave Rural Hospital for further management. The results of the blood investigations showed that the individual had been infected with the dengue virus (tested positive) and, on an MRI and CT scan, was diagnosed with an old case of subdural hematoma in the right frontotemporal region of the brain. The patient was admitted to the ICU for observation, where the child was not very responsive and had soft fontanelles with a decrease in the tone of the limbs. The patient developed symptoms suggestive of meningeal irritation, including continuous crying, refusal to feed, a stiff neck on examination, and multiple episodes of vomiting. The patient was on intravenous ceftriaxone and phenytoin. The child's head circumference was measured daily and showed gradual enlargement over two weeks. An evacuation of hematoma with a ventriculosubgaleal (VSG) shunt was planned and done in September 2023. The patient was under observation in the PICU after undergoing VSG shunting. In September 2023, physiotherapy rehabilitation was initiated as per the patient's consciousness. After four continuous weeks of intervention, the patient gained consciousness. In October 2023, physiotherapy rehabilitation was intended for poor neck control, a decrease in muscle tone, muscle weakness, speech, oromotor retraining, bed mobility, and balance and coordination.

Clinical findings

On examination, his vitals were stable, but he was non-responsive, having a Pediatric Glasgow Coma Scale (PGCS) of 8/15. The patient displayed symptoms of neck stiffness and meningeal irritation. On motor examination, the tone of the upper and lower limbs is measured on the Tone Grading Scale (TGS), where 0 is no response (flaccidity), 1+ decreased response (hypotonia), 2+ normal response, 3+ exaggerated response (mild to moderate hypertonia), and 4+ sustained response (severe hypertonia), as shown in Table 1. The reflex examination is shown in Table 2. Kernig’s and Brudzinski’s signs were present.

**Table 1:** Muscle tone of the upper and lower limbs

Muscle tone	Pre-rehabilitation		Post-rehabilitation	
	Right	Left	Right	Left
Shoulder muscles	2+	1+	2+	2+
Elbow muscles	2+	1+	2+	2+
Wrist muscles	2+	1+	2+	2+
Hip muscles	2+	1+	2+	1+
Knee muscles	2+	1+	2+	1+
Ankle muscles	2+	1+	2+	1+

**Table 2:** Reflexes examination

Reflexes	Biceps jerk	Triceps jerk	Supinator jerk	Knee jerk	Ankle jerk	Plantar response
Pre-rehabilitation						
Right	++	++	++	++	++	Flexor
Left	+	+	+	+	+	Absent
Post-rehabilitation						
Right	++	++	++	++	++	Flexor
Left	++	++	++	+	+	Flexor

Diagnostic assessment

**Figure 1:**
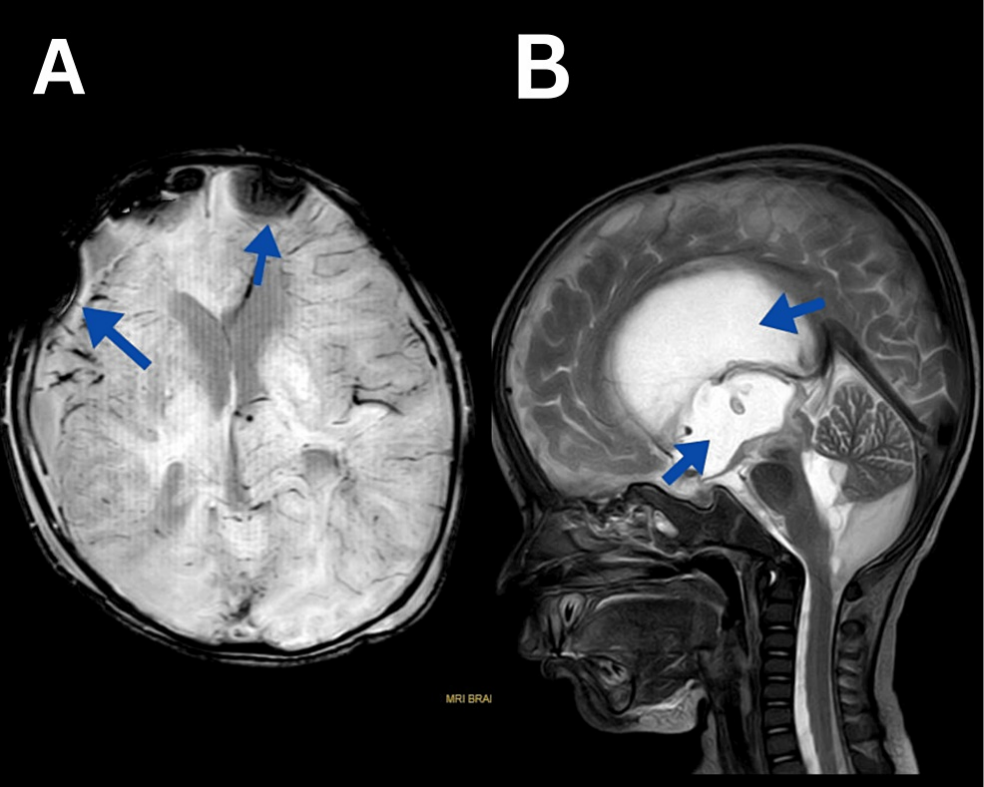
MRI findings of the brain (A) Subdural hemorrhage in the right frontal-temporal parietal and left frontoparietal region. (B) Hydrocephalus in the lateral, third ventricle, and fourth ventricle

A CBC revealed seropositive for (NS1 antigen) dengue virus infection, and on MRI, there is a subdural blood collection of a maximum thickness of 18 mm noted involving the right frontotemporal parietal regions. There is a thin strip of subdural blood collection with a maximum thickness of 3.3 mm indicated along the left front-parietal areas. There is communicating hydrocephalus noted in the form of moderate dilatation of bilateral lateral, third, and fourth ventricles, as shown in Figure 1.

Physiotherapy management

Plasticity plays a crucial role in the rehabilitation of individuals recovering from bacterial meningitis. This neurological phenomenon allows the brain to reorganize and form new connections, compensating for damaged areas. In meningitis, inflammation can affect cognitive and motor functions. Rehabilitation strategies leverage neuroplasticity to stimulate adaptive changes, promoting recovery. Targeted exercises, sensory stimulation, and cognitive tasks encourage the brain to rewire and regain lost functions. Plasticity-driven rehabilitation aims to optimize neural pathways, facilitating the restoration of cognitive and motor skills. This dynamic process underscores the importance of tailored interventions in promoting recovery and enhancing the quality of life for individuals affected by bacterial meningitis.

Physiotherapy rehabilitation primarily aimed at patient consciousness and secondary complications was planned for four weeks, as given in Table 3. Parents and caregivers were instructed to provide proper positioning and a constant change in positioning every two hours. Physiotherapy rehabilitation for neck control, speech, oromotor skills, hypotonia, bed mobility, and balance and coordination was planned after four weeks and continued for two months.

**Table 3:** Physiotherapy rehabilitation PNF: proprioceptive neuromuscular facilitation, reps: repetition

Problem identified	Goals	Treatment strategy	Intervention
Patient and family education	To enhance and maintain the patient's family's positive attitude towards treatment for early recovery	Interaction of therapist involving the patient and his family	The patient's mother, along with his family, was well-explained regarding his condition and was told about the importance of physiotherapy intervention
Consciousness	To stimulate sensory awareness	Sensory stimulation	Various forms of sensory input (visual, auditory, olfactory) can be used to stimulate the patient. Techniques like applying ice, using textured materials, playing music, or providing familiar scents
	To help the patient become more aware of their own body	Proprioceptive and tactile stimulation	Roods approach (tapping on the belly and tendon)
			Applying pressure to the patient's limbs or using techniques like rubbing the skin to stimulate receptors below the skin
	To stimulate the vestibular system	Vestibular stimulation	Techniques like gentle rocking or slow tilting of the patient's head and body
Prevention of pressure sores	To prevent bed sores	Positioning	Frequent repositioning of the patient to avoid pressure sores and improve comfort, positioning using pillows
Lack of deglutition, speech, and overall oromotor skills	To improvise deglutition and speech	Oromotor retraining	Oral sensory stimulation, lip and tongue exercises (lip pursing, tongue protrusion, lateral tongue movement), oral motor massage, facial and oral exercises
Poor neck control	To improve neck control	Neurodevelopmental techniques	Facilitation on the spine in the prone position on a bolster or ball
Hypotonia	To enhance muscular tone and stimulate and re-educate the muscles, improving tone and control	Facilitation techniques for tone development	PNF Rhythmic Initiation for upper and lower limb (D1 and D2 patterns) (10 reps x 1 set)
			Joint approximation (10 rep x 1 set)
Unable to perform bed mobility independently	To promote bed mobility	Functional mobility activities	Mat exercises (supine to-side lying and side lying-to-sitting activity performed)
Balance and coordination impairment	To gain static and dynamic balance	Balance and coordination retraining	Weight-bearing in a quadruped position and multidirectional reach-outs in sitting

**Figure 2:**
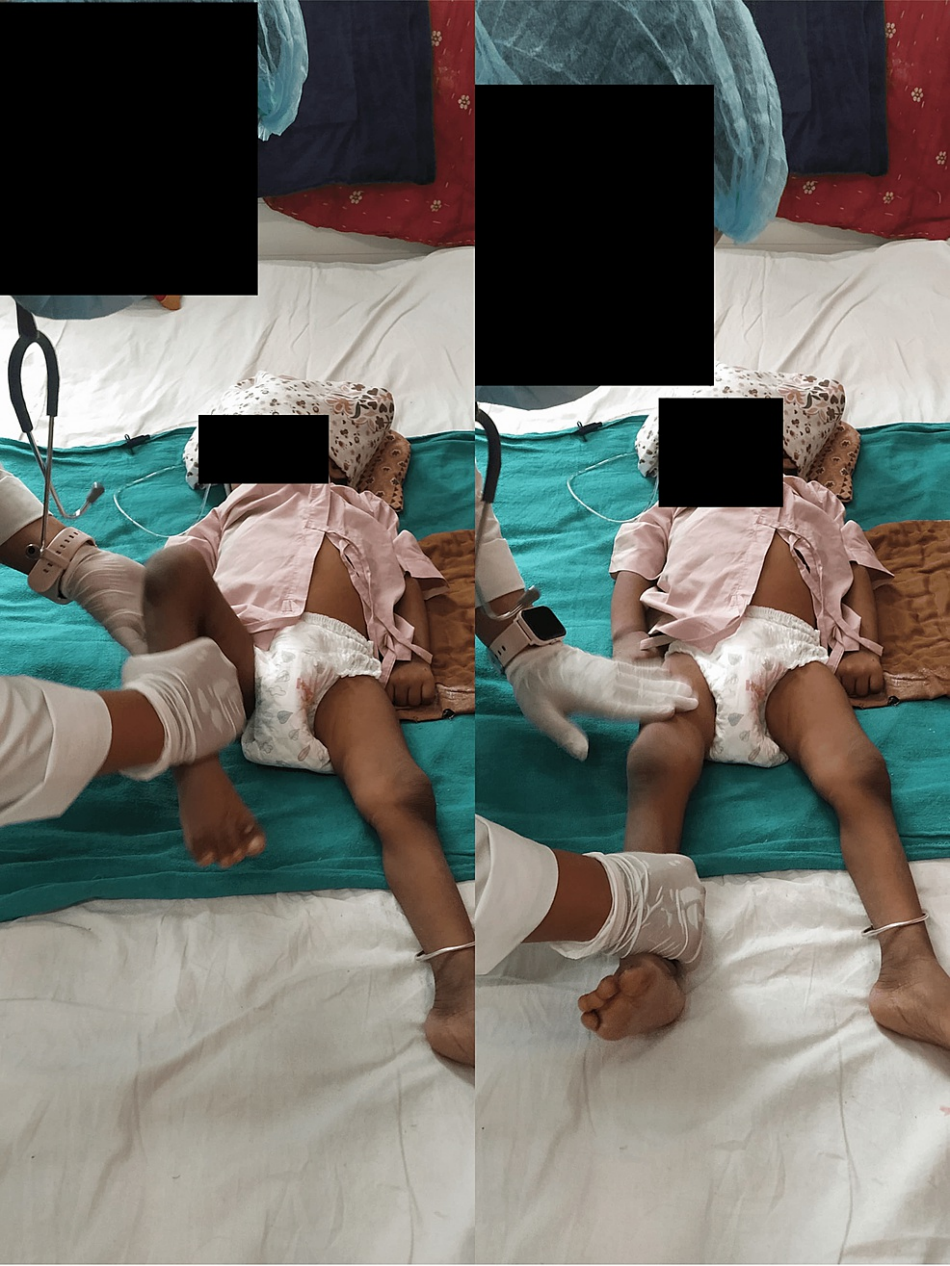
Roods approach (tapping) for the right lower limb

**Figure 3:**
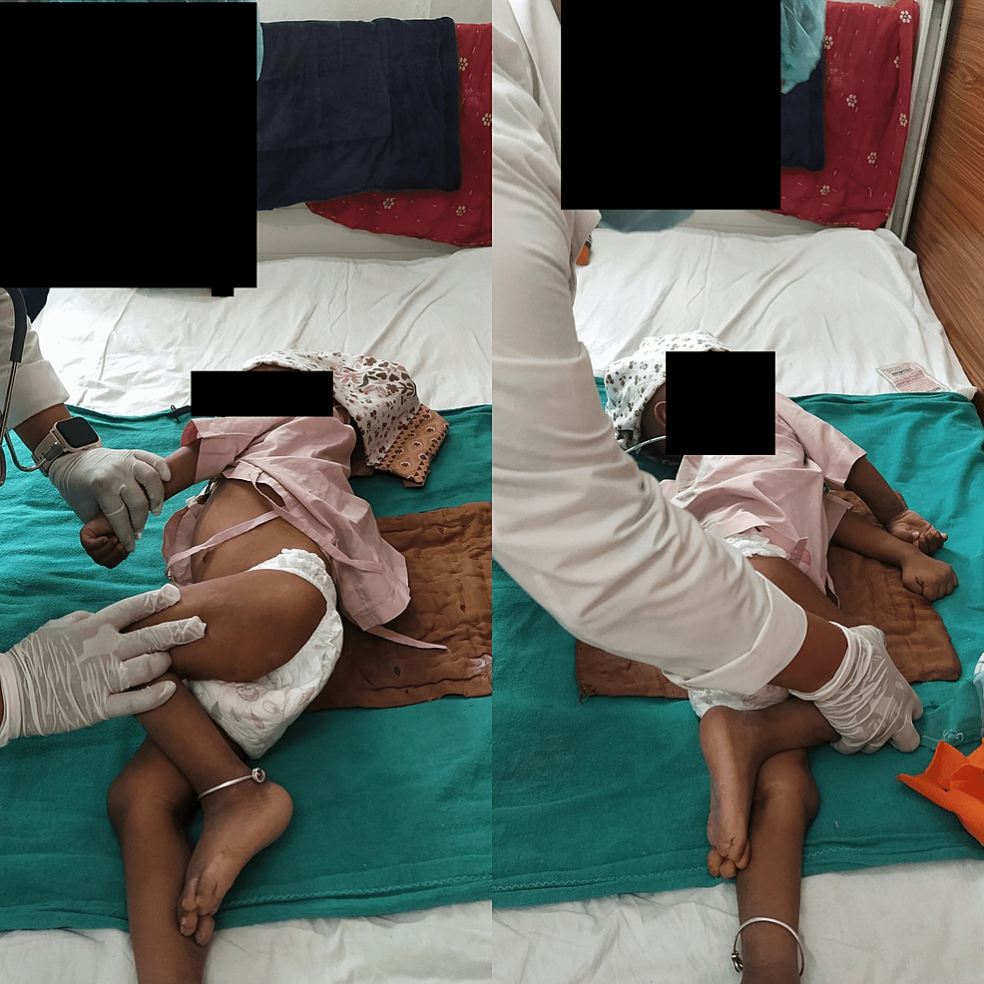
Bed mobility exercise side-rolling to the right and left sides

The physiotherapy intervention received by the patient is shown below (Figure 2). It shows the therapist giving the Roods approach (tapping) to the right upper limb. Figure 3 shows the therapist giving bed mobility exercises side-rolling to the right and left sides.

Follow-up and outcome measures

An organized physical therapy interventional protocol was started with follow-up after every four weeks. After four continuous weeks of neurophysiotherapy intervention, including sensory stimulation, vestibular stimulation, and proprioceptive and tactile stimulation, the child gained consciousness. There was reduced head control, decreased muscle tone, and delayed developmental milestones, which were achieved until 18 months of age. The baby's physiotherapy plan aims to resolve all the issues mentioned within eight weeks.

The PGCS rates three components: motor reaction (M), verbal response (V), and eye-opening response (E), on a scale of 1 to 6. The score ranges from 3 to 15, with higher values indicating greater consciousness. Based on a theoretical range of 0 to 16, the Full Outline of UnResponsiveness (FOUR) score is a 17-point scale. Lowering FOUR scores is linked to a declining state of awareness. Eye responses, motor responses, brainstem reflexes, and breathing patterns are the four neurological function domains evaluated by the FOUR Score.

The Functional Independence Measure for Children (WeeFIM) is an instrument consisting of 18 items arranged in seven levels, measuring a child's consistent performance in fundamental everyday functioning skills. During evaluations, a child's self-care, mobility, and cognition are assessed through interviews and task completion based on specific criteria. The two primary functional streams of WeeFIM are "dependent" (i.e., needs assistance; scores 1-5) and "independent" (i.e., requires no service; scores 6-7). The findings of the outcome measure are shown in Table 4.

**Table 4:** Outcome measures pre- and post-scores PGCS: Pediatric Glasgow Coma Scale, FOUR: Full Outline of UnResponsiveness, TGS: Tone Grading Scale, WeeFIM: Functional Independence Measure for Children

Sr no.	Outcome measures	Pre-score	Post-score
1.	PGCS	8/15	13/15
2.	FOUR score	5/16	12/16
3.	TGS	1/4	2/4
4.	WeeFIM	1/7	5/7

## Discussion

Timely diagnosis and patient stabilization are critical for the successful treatment of meningitis patients. For a hemodynamically unstable patient, practitioners should first concentrate on establishing venous access and starting supportive therapy [13]. Since encapsulated bacteria are the primary cause of bacterial meningitis, vaccinations against these bacteria have dramatically decreased the incidence in developed nations. As a result, cases of bacterial meningitis can occasionally be clinically unsure, as can other sick children with febrile illnesses [14]. Meningitis can progress to subdural empyema, particularly in the 12-20 month age group when little is known about the disease's features. Long-term neurologic consequences and death may result from delayed diagnosis and treatment [15].

Dunbar et al. conducted a study in which they found that acute bacterial meningitis and a clinically justified MRI were associated with more than one-third of children having an ischemic stroke. The length of the disease, seizures, and the causative organisms were all linked to stroke as clinical variables [16]. Seizures are more likely in infants from three months to one year of age during acute bacterial meningitis. Those diagnosed with focal or secondary generalized seizures had worse outcomes. Hospitalized patients with seizures had abnormal neuroimaging. Late seizures were more likely than acute seizures [17].

The caregiver's consistency in providing home exercise routines and holding multiple sessions to address the mother's worries was also crucial to the treatment regimen's success. We witnessed consistent progress every week. After a month of therapy, the kid was able to stand with assistance while sitting, maintain balance while sitting, and initiate reach-outs while sitting. After six weeks, the child's caregivers were ecstatic to see him walk with little assistance and good trunk and pelvic control [18]. Montgomery et al. conducted a longitudinal case report, "Achievement of Gross Motor Skills in Two Children with Cerebellar Hypoplasia," and concluded that this information may be valuable for physical therapists and families dealing with similar diagnoses and clinical symptoms, aiding in the prediction of motor outcomes and the planning of interventions [19]. Mohamed et al. conducted a study, "Efficacy of Physiotherapy and Conductive Education in Improving Motor Skills and Mental Function in Children With Cerebral Palsy," in which they concluded that following six months of consistent physiotherapy and a conductive education program, three groups showed significant improvements in fine motor, gross motor, and cognitive abilities. We had greater results with early PT start times and conductive education programs [20].

## Conclusions

Based on a case report, it has been concluded that early integrative neurophysiotherapy can be effective in improving gross motor developmental milestones in children with meningitis. The therapy involves a goal-oriented therapeutic regimen that includes various stimulation techniques, positioning, oromotor retraining, neurodevelopmental techniques, proprioceptive neuromuscular facilitation techniques, mobility exercises, and balance and coordination retraining. After undergoing therapy, the child in this case showed significant clinical improvement as well as improvement in outcome measures. Within a month of starting therapy, the child was able to sit with minimal assistance and, within two months, achieved independent sitting. The child also demonstrated good muscle tone and balance while sitting after completing the rehabilitation program. Engaging patients in immersive exercises that enhance neuroplasticity and accelerate cognitive and motor recovery with interactive stimuli is a novel approach.
